# ROBOTIC ASSISTED SINGLE SITE FOR BILATERAL INGUINAL HERNIA REPAIR

**DOI:** 10.1590/0102-6720201600020011

**Published:** 2016

**Authors:** Henrique Rasia BOSI, José Ricardo GUIMARÃES, Leandro Totti CAVAZZOLA

**Affiliations:** Department of General Surgery, Hospital de Clínicas de Porto Alegre, Porto Alegre, RS, Brazil

**Keywords:** Hernia, inguinal, Robotics, Laparoscopy, General surgery

## Abstract

**Background:**

The inguinal hernia is one of the most frequent surgical diseases, being frequent procedure and surgeon´s everyday practice.

**Aim:**

To present technical details in making hernioplasty using robotic equipment on bilateral inguinal hernia repair with single port and preliminary results with the method.

**Method:**

The bilateral inguinal hernia repair was performed by using the Single-Site^(c)^ Da Vinci Surgical Access Platform to the abdominal cavity and the placement of clamps.

**Results:**

This technique proved to be effective for inguinal hernia and have more aesthetic result when compared to other techniques.

**Conclusions:**

Inguinal hernia repair robot-assisted with single-trocar is feasible and effective. However, still has higher costs needing surgical team special training.

## RESUMO

**Racional:**

A hérnia inguinal é uma das doenças cirúrgicas mais frequentes, tornando-a procedimento frequente e do cotidiano do cirurgião.

**Objetivo:**

Apresentar detalhes da técnica da hernioplastia inguinal bilateral robótica por single-site e resultados preliminares com o método.

**Método:**

Foi realizada hernioplastia inguinal bilateral assistida por robô, utilizando-se da Vinci Single-Site(c) Surgical Platform para acesso a cavidade abdominal e colocação das pinças.

**Resultados:**

Esta técnica demonstrou-se efetiva para correção da hérnia inguinal, além de apresentar melhor resultado estético quando comparado às outras técnicas.

**Conclusões:**

A hernioplastia inguinal assistida por robô com trocarte único é viável e eficaz. Contudo, ainda apresenta custos mais elevados e necessidade de treinamento especial por parte da equipe cirúrgica.

## INTRODUCTION

The groin is the most frequent site affected by hernias in the abdominal wall. It is estimated that its incidence is 100-300 cases per 100,000 inhabitants per year, turning inguinal hernia surgery in one of the most commonly abdominal operations performed today. The laparoscopic repair began in the early 1990^5^ and since then, have become increasingly popular. Because it causes less metabolic response to trauma, this will result in less pain in postoperative period, providing rapid return of patients to their activities^7^. Considering laparoscopy for inguinal hernia repair, the surgeon can choose the total extraperitoneal (TEP) or trans-abdominal preperitoneal (TAPP)^1^.

The increased demand for postoperative aesthetic results led to raise interest in the operation with a single portal (LESS - Laparoendoscopic single site surgery)^6^. The main technical problems encountered with the use of this technique were the loss of instruments triangulation and the collision of tweezers^4^. However, with the new instruments and ports for access to the abdominal cavity, LESS started to show greater application in surgical specialties^2^.

The robotic platform added to laparoscopy the enrichment of movement, the ease of maneuvers and procedures, the view in three dimensions and the ergonomics for the surgeon. In 2010, it was created the Da Vinci Single-Site^(c)^ Surgical Platform (DVSSP), allowing to perform robotic procedures with this platform, which adds the advantages of robotic surgery to LESS procedures, allowing greater triangulation caused by the characteristics of the system.

This study reports the robotic technique for robotic assisted single site bilateral inguinal hernia repair, describing the technique used for the completion and preliminary results with the method.

## METHOD

### Technique

**FIGURE 1:**
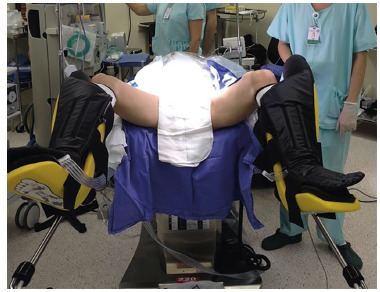
Patient positioning

Patient was positioned in supine and Trendelenburg position. Antibiotic prophylaxis with cefazolin was given (Figure 1). Leggings were used and the robot was coupled ("docking") through the distal end of the patient.

**FIGURE 2:**
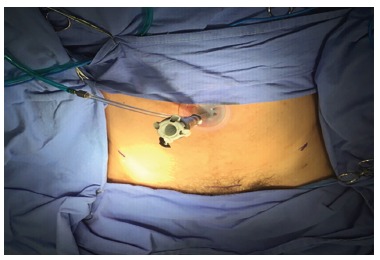
Single site positioning: the side dots demarcate the anterior superior iliac spines and the lower the pubis

 A 25 mm incision was held in the umbilical region for insertion of the specific robotic single site trocar, which is characteristic by cross entry of tweezers (instrument entering the left side of the portal is the effector of the patient's right and vice versa) (Figure 2). The robotic software allows the surgeon to the intuitive control of his hand, that is, the hand that is in use controls the instrument ipsilaterally, no matter the tweezer entrance point. The instrument that appears to the right on the screen is controlled by the right surgeon control in the robotic console.

The hook for cauterization with monopolar energy was placed laterally to the side of dissection and also a robotic Mariland in medial position. During the procedure the camera used was 30° optics, driven down. The peritoneum was marked with cautery, starting from the rear and lateral side. The preperitoneal space was dissected, exposing the deep inguinal ring, inferior epigastric vessels making possible the evaluation of the defect. A polypropylene mesh was used, measuring 10x15 cm, covering all possible inguinal defects. The mesh was fixed using Protack 5 mm stapler with titanium non-absorbable staples, placed in specific robotic single site port used by auxiliary instruments. The pre-peritoneal space was also closed with staples. The console time was approximately 90 minutes.

**FIGURE 3:**
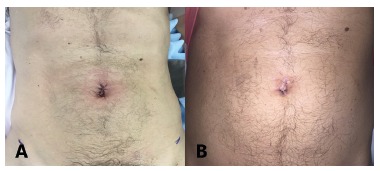
A) Immediate postoperative; B) 10th postoperative day

 After the procedure, the robot was uncoupled ("undocking") and the umbilical port was removed. The closure of umbilical aponeurosis was performed with Vicryl(r)-0 in continuous suture, using the technique of "small bites", and the skin with Mononylon(r) 4-0 (Figure 3). 

## RESULTS

This method was used in a man, 62-year-old, ex-smoker, BMI 26 kg/m^2^, complaining of pain in bilateral groin region, mainly on the left. On physical examination, the abdomen revealed no particularities, with the presence of reducible bulging in bilateral inguinal region and worsens with the Valsalva maneuver.

After obtaining surgical and anesthetic consent term, the patient was referred to the operating room, reassessing the defect that was bilateral.

**FIGURE 4:**
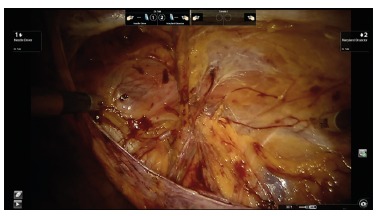
Dissection of pre-peritoneal space

**FIGURE 5:**
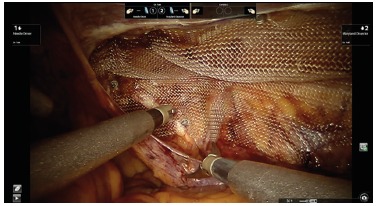
Mesh fixation

**FIGURE 6:**
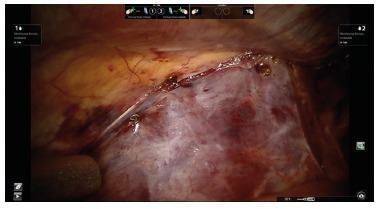
Closing the pre-peritoneal space

The robotic assisted single site hernioplasty was performed with the Da Vinci Single-Site^(c)^. The patient was placed in the Trendelenburg position with leggings, under general anesthesia. The robotic equipment was placed at the caudal end. After the introduction of the optics, the trocar was placed under direct vision and then, performed the "docking" with the help of the nursing staff. The surgery followed the concepts of laparoscopic technique (Figures 4, 5 and 6). The advantage of the robotic platform is to allow larger movements and better ergonomics for the surgeon when compared to non-robotic laparoscopy single site. The tridimensional visualization allows more delicate dissection of the structures, turning the procedure more precise. This technique proved to be safe and effective, helping to solve the limitation found in conventional laparoscopy.

At the end of the procedure, the patient was referred to the post-anesthesia care unit, receiving intravenous analgesia. The patient had hospital discharged in first postoperative day. Outpatient follow up in 10 days, he was asymptomatic without any complication.

## DISCUSSION

The surgical treatment of groin hernia has undergone many changes since its first descriptions. The techniques may be divided into two groups: anterior repair and pre-peritoneal, being the latter used by laparoscopic techniques. 

Laparoscopic herniorrhaphy became popular from the 1990s. Meta-analysis of the Cochrane in 2013 compared the open with laparoscopic technique, demonstrating that this one, despite causing major complication that rarely occur in conventional repair (intestinal perforation and major vessels injury), promotes earlier return to daily activities, lower incidence of chronic pain and similar recurrence rate^8^. Aiming to better surgical and aesthetic results, less invasive procedures increasingly began to be used.

Laparoscopic surgery with single site has gained popularity. However, some technical challenges still need to be overcome, such as the loss of triangulation and the instruments collision. Moreover, it has a higher learning curve for a surgeon^4^.

Robotic surgery offers better visualization and more precise movements, thereby, reducing the tissue trauma and the likelihood of postoperative neuralgia. Currently, the use of single site can be associated with robotic technology. In a series of 34 cases^3^, the average time of the unilateral non-complicated inguinal hernia surgery was 69 min, been less than the average time found in laparoscopic single site technique (96 min)^4^.

The selection of patients appears to interfere with the success of the technique. Patients with BMI greater than 30 kg/m^2^ may bring technical limitations. However, there are no reports of conversion with multiple port or open. Potential complication is the herniation in the port site^3^, which is the subject of debate and conflicting results in the literature. 

The case reported here is the first procedure of its kind held in Brazil with the help of robotics platform. Prospective studies are needed to evaluate the advantages or disadvantages, comparing this with other accepted methods for the treatment of groin hernias in long-term follow up.

## CONCLUSION

The robotic assisted single site hernia repair is feasible and effective. However, still has higher costs and the need for special training by the surgical team. 
